# The iron chelator Dp44mT inhibits hepatocellular carcinoma metastasis via N-Myc downstream-regulated gene 2 (NDRG2)/gp130/STAT3 pathway

**DOI:** 10.18632/oncotarget.2328

**Published:** 2014-08-08

**Authors:** Jiabei Wang, Dalong Yin, Changming Xie, Tongsen Zheng, Yingjian Liang, Xuehui Hong, Zhaoyang Lu, Xuan Song, Ruipeng Song, Haiyan Yang, Boshi Sun, Nishant Bhatta, Xianzhi Meng, Shangha Pan, Hongchi Jiang, Lianxin Liu

**Affiliations:** ^1^ Department of Hepatic Surgery, The First Affiliated Hospital of Harbin Medical University, Key Laboratory of Hepatosplenic Surgery, Ministry of Education, Harbin, Heilongjiang Province, China; ^2^ Department of Pharmacology (the State-Province Key Laboratories of Biomedicine-Pharmaceutics of China, Key Laboratory of Cardiovascular Research, Ministry of Education), Harbin Medical University, Harbin, China

**Keywords:** Dp44mT, NDRG2, hepatocellular carcinoma, metastasis, STAT3

## Abstract

Here we showed that hepatocellular carcinoma (HCC) cell lines with high metastatic potential had low levels of NDRG2. The iron chelator Dp44mT up-regulated NDRG2, suppressed epithelial-mesenchymal transition (EMT) and inhibited tumor metastasis in HCC having high metastatic potential. Also Dp44mT attenuated the TGF-β1-induced EMT in HCC having low metastatic potential. In agreement, silencing endogenous NDRG2 with shNDRG2 in HCC cells attenuated the effect of Dp44mT. We showed that the NDRG2/gp130/STAT3 pathway can mediate Dp44mT effects. In agreement, we found that a combination of NDRG2 expression and p-STAT3 levels is a strong predictor of prognosis in HCC patients. We suggest that up-regulation of NDRG2 by Dp44mT is a promising therapeutic approach in HCC.

## INTRODUCTION

Hepatocellular carcinoma (HCC) is the third leading cause of cancer death worldwide, and the second in China.[1] Although resection is considered as a potentially curative treatment for HCC patients, the five-year postoperative survival rate is 30% to 40%.[2] The extremely poor prognosis of patients with HCC is largely due to the high frequency of tumor recurrence or distant metastasis.[3, 4] However, the molecular mechanism underlying HCC metastasis remains unclear. Therefore, the identification of novel anticancer drugs and molecular markers will provide new opportunities for the prevention of HCC recurrence and metastasis.

Iron is essential for both normal and cancer cells. It is required by many proteins involved in cell growth and proliferation. Neoplastic cells have an increased requirement for iron as shown by their markedly elevated expression of the transferrin receptor 1 and enhanced uptake of iron.[5] Several studies showed excess iron has carcinogenicity, and iron reduction could prevent carcinogenesis in a supposedly normal population.[6-10] However, the precise molecular pathways involved remain unclear and are important to elucidate particularly in terms of the mechanisms involved in metastasis, which is a major problem in cancer treatment.

Iron chelators are a relatively new class of potential anti-metabolites that show marked and selective anti-tumor activity,[5, 11] although their molecular targets and mechanisms of action remain to be completely elucidated. To date, the most widely used chelator in clinical settings for the treatment of iron overload is desferrioxamine (DFO), and its antiproliferative activity against neuroblastoma and leukemia has been examined in clinical trials.[5] However, the use of DFO as an anticancer agent is limited by its modest antiproliferative activity that is related to its poor membrane permeability and short half-life.[12, 13] In contrast, novel thiosemicarbazone chelators, such as di-2-pyridylketone-4,4-dimethyl-3-thiosemicarbazone (Dp44mT), demonstrated far greater antiproliferative activity and Fe chelation efficacy than DFO and showed promise as an antitumor agent.[14, 15] This chelator belongs to the di-2-pyridyl thiosemicarbazone (DpT) group, which shows high affinity and selectivity for Fe(III).[15]

NDRG2 belongs to the NDRG (N-myc downstream-regulated genes) family where it has been reported to function as a tumor and metastasis suppressor gene.[16-18] NDRG2 (mRNA and protein) has been described to be significantly downregulated in a variety of human cancer cell lines and primary tumors. NDRG2 promoter CpG island methylation and down-regulation have been observed in breast,[19] colon,[20] and lung cancer cell lines[19] as well as primary glioblastomas[21] and meningiomas,[22] liver,[23] and colorectal cancer (CRC).[20] Hence, NDRG2 will be a promising molecular target for cancer therapy that could be modulated by novel iron chelators. However, the detailed mechanisms for the anti-cancer effects of NDRG2 are not well elucidated and further investigation is required.

In this study, we extensively investigated the function of Dp44mT and determined its contribution to inhibiting HCC invasion and metastasis. We also dissected the molecular mechanisms by which Dp44mT mediates tumor metastasis. Results presented here suggested that Dp44mT inhibits HCC aggression via up-regulation of NDRG2. NDRG2 inhibits HCC metastasis via modulation of gp130/STAT3 signaling. We propose that combination of NDRG2 and STAT3 is a new powerful predictor for HCC recurrence and metastasis and a new potential target for adjuvant treatment of aggressive HCCs after surgical resection.

## RESULTS

### Low expression of NDRG2 in HCC cell lines with metastatic potential and in invasive HCC specimens

**Figure 1:**
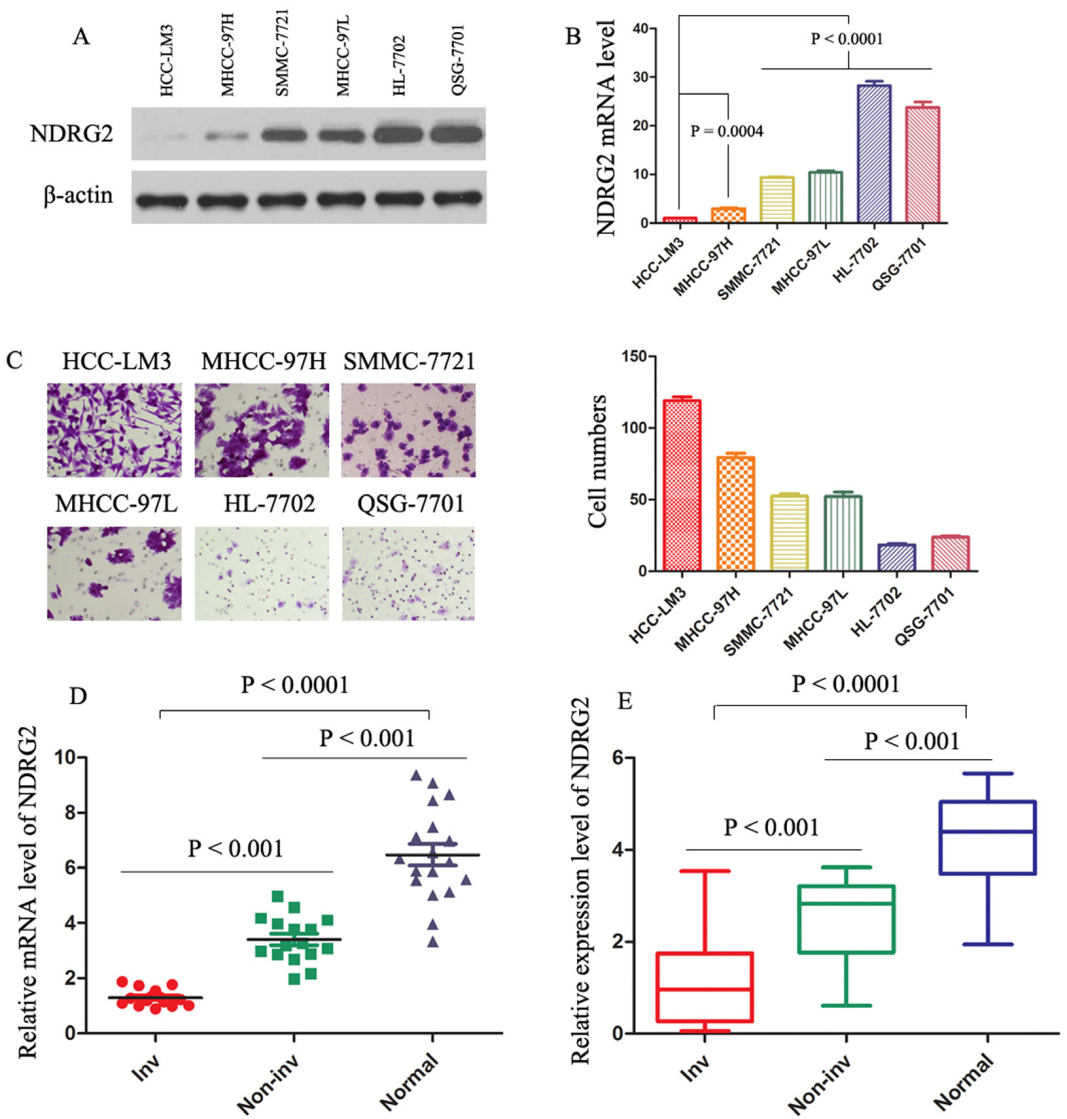
Levels of NDRG2 are decreased in HCC clinical samples, and decreased levels of NDRG2 indicate invasion/metastasis of HCC (A) and (B) The protein and mRNA levels were evaluated in the indicated cell lines. The expression of NDRG2 was normalized against β-actin. (C) Cell invasion assay was performed in the indicated cells. The invading cells through the ECM were enumerated 48 hours later. The results represent means ± SD of experiments performed in triplicate. (D) Real-time PCR results of relative expression level of NDRG2 mRNA in 17 normal liver (Normal), 16 HCC without vascular invasion (Non-inv), and 15 HCC with vascular invasion (Inv) samples. mRNA levels of NDRG2 were normalized against β-actin. The results represent means ± SD of experiments performed in triplicate. (E) Protein levels of NDRG2 were determined in 15 Normal, 15 Non-inv, and 15 Inv samples. Box plot graph showed the statistical analysis of NDRG2 expression in all samples. The expression of NDRG2 was normalized against β-actin.

We first examined the NDRG2 protein amounts in several HCC cell lines, with varying metastatic capability.[24, 25] The NDRG2 protein levels decreased progressively from normal liver cells (HL-7702 and QSG-7701), low metastatic SMMC-7721 and MHCC-97L cells, to highly metastatic MHCC-97H and HCC-LM3 cells (Fig. 1A). Real-time polymerase chain reaction (PCR) analysis showed the same pattern of NDRG2 mRNA level (Fig. 1B). Cell invasion assay was performed in the HCC cell lines mentioned above (Fig. 1C). Low expression of NDRG2 was significantly associated with higher invasive capacity of MHCC-97H and HCC-LM3 cells as compared with the other four HCC cell lines. Real-time PCR analysis showed that NDRG2 expression was significantly lower in invasive HCC samples than in normal liver tissue or noninvasive HCC tumors (Fig. 1D). Immunoblotting of protein extracted from the same set of patients' samples confirmed the association of NDRG2 down-regulation with features of tumor metastasis (Fig. 1E; Supporting Information Fig. 1A), suggesting NDRG2 involvement in HCC aggressiveness. Intriguingly, Table 1 also showed the NDRG2 expression levels were found to be significantly lower in HCCs with microvascular invasion (*P* < 0.0001), TNM stage (II-III) (*P* = 0.033), Edmonson grade (III-IV) (*P* = 0.018) and infiltrative growth pattern (*P* = 0.045).

**Table 1:** Correlation between NDRG2 staining and clinicopathologic characteristics in 136 HCC patients

Variabls	NDRG2 staining		
	High (n=55)	Low (n=81)	
	n(%)	n(%)	P value
Age (years)			
≤50	26(47.27%)	44(54.32%)	0.420
>50	29(52.73%)	37(45.68%)	
Gender			
Male	42(76.36%)	67(82.72%)	0.362
Female	13(23.64%)	14(17.28%)	
HBsAg			
Negative	11(20.00%)	15(18.52%)	0.830
Positive	44(80.00%)	66(81.48%)	
Anti-HCV			
Negative	53(96.36%)	77(95.06%)	0.717
Positive	2(3.64%)	4(4.94%)	
Liver cirrhosis			
No	9(16.36%)	17(19.32%)	0.501
Yes	46(83.64%)	64(80.68%)	
Serum AFP, ng/ml			
≤20	15(27.27%)	20(24.69%)	0.735
>20	40(72.73%)	61(75.31%)	
Tumor diameter (cm)			
≤5	35(63.64%)	41(50.62%)	0.133
>5	20(36.36%)	40 (49.38%)	
Microvascular invasion			
No	40(72.73%)	32(39.51%)	0.0001
Yes	15(27.27%)	49(60.49%)	
Tumor number			
Single	37(67.27%)	48(59.26%)	0.344
Multiple	18(32.73%)	33(40.74%)	
TNM stage			
I	34(61.82%)	35(43.21%)	0.033
II -III	21(38.18%)	46(56.79%)	
Edmonson grade			
I-II	45(81.82%)	51(62.96%)	0.018
III-IV	10(18.18%)	30(37.04%)	
Growth pattern			
Expansile	49(89.09%)	61(75.31%)	0.045
Infiltrative	6(10.91%)	20(24.69%)	

Abbreviations:AFP, alpha-fetoptotein; HBsAg, hepatitis B surface antigen; HCV, hepatitis C virus.

### Dp44mT inhibits the invasive potential of HCC cells *in vitro*

**Figure 2:**
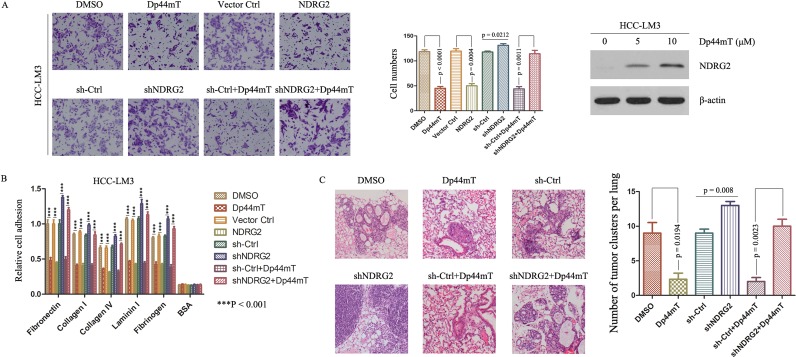
Dp44mT inhibits invasion and adhesion of cancer cells *in vitro* and inhibits metastasis *in vivo* through up-regulating NDRG2 (A) Dp44mT increased the expression of NDRG2 significantly in HCC-LM3 cells. Cell invasion experiment results showed Dp44mT (10 μM) or NDRG2 overexpression significantly inhibited the invasive capacity of HCC-LM3 cells, and NDRG2 knockdown attenuated the Dp44mT-induced inhibition of invasion in HCC-LM3 cells. The results represent means ± SD of experiments performed in triplicate. (B) 24 hours after Dp44mT (10 μM) treatment, adhesion to matrix proteins were performed in HCC-LM3 cells (which infected with NDRG2 or shNDRG2). Adhering cells were quantified by CytoSelectTM Cell Adhesion Assay Kit. The results represent means ± SD of experiments performed in triplicate. (C) 4 weeks after Dp44mT treatment, lung tissues were harvested. Representative lung tissue sections from each group were shown (hematoxylin and eosin stain; magnification, × 40). The number of lung metastatic foci in each group was calculated.

To determine the function of Dp44mT, we treated HCC-LM3 and MHCC-97H with Dp44mT. Dp44mT significantly inhibited their invasive capacity by 2.5- and 2.3-fold, as compared with DMSO-treated cells, and enhanced the expression of NDRG2 (Fig. 2A; Supporting Information Fig. 2A). Dp44mT also inhibited HCC-LM3 and MHCC-97H's adhesion to several cell matrix proteins (Fig. 2B; Supporting Information Fig. 2B). In contrast, lentivirus mediated silencing endogenous NDRG2 in HCC-LM3 and MHCC-97H cells attenuated the Dp44mT-induced inhibition of invasion (Fig. 2A; Supporting Information Fig. 2A), and resulted in promotion of adhesion to cell matrix proteins (Fig. 2B; Supporting Information Fig. 2B). In order to determine the significance of NDRG2, retrovirus mediated transfection of human NDRG2 into HCC-LM3 and MHCC-97H cells was performed. NDRG2 overexpression also inhibited their invasive capacity (Fig. 2A; Supporting Information Fig. 2A). Supporting Information Figure 1B showed the expression of Flag and NDRG2 after transfection. Evidently, Dp44mT acted to inhibit the invasive property of HCC cells via up-regulation of NDRG2 *in vitro*.

### Dp44mT inhibits metastasis of HCC *in vivo*

We further examined the effect of Dp44mT on HCC pulmonary metastasis by establishing an orthotopic liver tumor model in nude mice. HCC-LM3 cells having high metastatic potential were used for orthotopic model studies. Compared to DMSO groups, Dp44mT treatment resulted in significant decrease of the number of pulmonary metastatic foci and average size of pulmonary metastatic lesions (Fig. 2C). Furthermore, the orthotopic liver tumor model based on high-metastatic potential MHCC-97H cells also showed that NDRG2 overexpression inhibited lung metastasis (Supporting Information Fig. 2C). On the contrary, down-regulation of NDRG2 expression by shNDRG2 attenuated the effect of Dp44mT-induced reduction of pulmonary metastases dramatically (Fig. 2C). Together, these results revealed functional significance of Dp44mT with high propensity to inhibit metastasis in metastatic HCC and in aggressive tumors.

### Dp44mT inhibits tumor metastasis by suppressing epithelial- mesenchymal transition (EMT) in HCC having high metastatic potential

**Figure 3:**
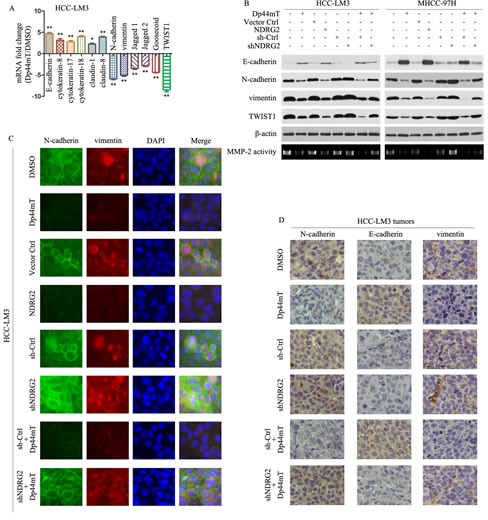
Dp44mT inhibits EMT in HCC cells (A) 12 hours after Dp44mT (10 μM) treatment, real-time PCR was performed to assess mRNA levels of epithelial markers E-cadherin, cytokeratin-8, cytokeratin-17, cytokeratin-18, claudin-1, and claudin-8 and mesenchymal markers N-cadherin, vimentin, Jagged1, Jagged2, Goosecoid, and an EMT regulator TWIST1 in HCC-LM3 cells. Results were normalized against β-actin. The results represent means ± SD of experiments performed in triplicate. (B) 24 hours after treatment, immunoblotting showed Dp44mT (10 μM) or NDRG2 overexpression increased the expression of E-cadherin and decreased the expression of N-cadherin, vimentin and TWIST1 in HCC-LM3 and MHCC-97H cells. Knockdown of NDRG2 abrogated the effect of Dp44mT-induced reduction of EMT. β-actin was used as the internal control. All assays were done in triplicate. (C) Single and merged images were taken to show immunofluorescence staining of N-cadherin (green) and vimentin (red) accompanied by the cell nucleus (blue) stained by DAPI. (D) HCC-LM3 liver tumors from different groups were immunostained for indicated molecules. Pictures are representative of three independent experiments.

Given that Dp44mT inhibited HCC metastasis, we investigated the effect of Dp44mT on EMT, a critical event in tumor invasion. We performed real-time PCR for molecular markers of EMT. The epithelial markers such as E-cadherin, cytokeratin-8, cytokeratin-17, cytokeratin-18, claudin-1, and claudin-8 were higher in the Dp44mT group than that in the DMSO group, whereas the mesenchymal markers such as N-cadherin, vimentin, Jagged 1, Jagged 2, Goosecoid, and the EMT major regulator TWIST1 were decreased (Fig. 3A). Immunoblotting also detected higher expression of E-cadherin in HCC-LM3 and MHCC-97H cells which treated with Dp44mT. In contrast, the expression of N-cadherin, vimentin, and TWIST1 decreased in Dp44mT treated HCC-LM3 and MHCC-97H cells (Fig. 3B). Gelatin zymography assay showed that matrix metalloproteinase-2 (MMP2) activity was decreased in Dp44mT treated HCC-LM3 and MHCC-97H cells (Fig. 3B). NDRG2 overexpression in HCC cells had similar effect with Dp44mT treatment, and down-regulation of NDRG2 expression by shNDRG2 abrogated the effect of Dp44mT-induced reduction of EMT(Fig. 3B). Immunofluorescence also showed Dp44mT and NDRG2 overexpression can increase the expression of E-cadherin, decrease the expression of N-cadherin and vimentin in HCC-LM3 and MHCC-97H cells, shNDRG2 can abrogate the effect of Dp44mT (Fig. 3C, Supporting Information Fig. 3A and 4). We next investigated the occurrence of EMT *in vivo*. As analyzed by immunohistochemistry, in HCC-LM3 tumors, Dp44mT treatment exhibited the inhibition of typical EMT phenotype, including focal increment of the epithelial marker E-cadherin and concurrent loss of the mesenchymal marker vimentin and N-cadherin. And shNDRG2 can abrogate the effect of Dp44mT (Fig. 3D). In MHCC-97H tumors, NDRG2 overexpression had similar effect with Dp44mT treatment (Supporting Information Fig. 3B).

### Dp44mT attenuates the TGF-β1-induced EMT in HCC having low metastatic potential

**Figure 4:**
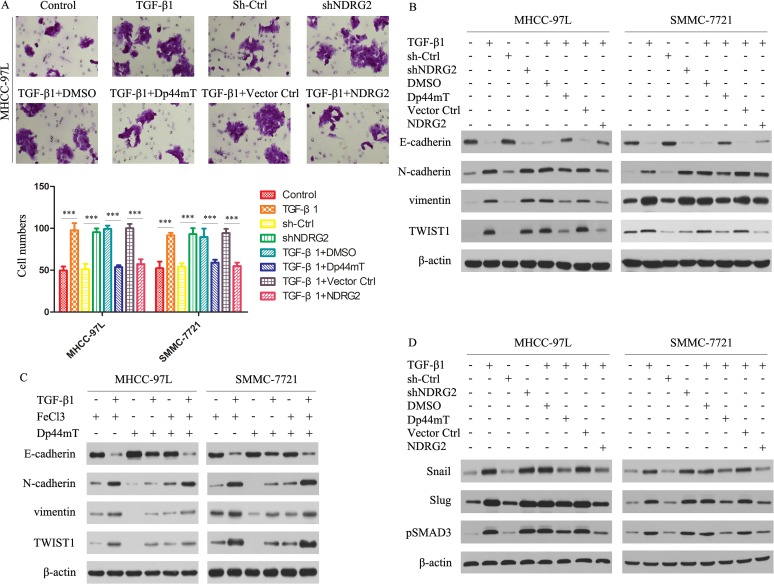
Dp44mT attenuates the TGF-β1-induced EMT in MHCC-97L and SMMC-7721 cells (A) Cell invasion experiment results showed TGF-β1 (5 ng/ml) or NDRG2 knockdown significantly enhanced the invasive capacity of MHCC-97L cells, and Dp44mT (10 μM) and NDRG2 overexpression attenuated the TGF-β1/ NDRG2 knockdown induced activation of invasion in MHCC-97L and SMMC-7721 cells. The results represent means ± SD of experiments performed in triplicate. (B) Cells were pretreated in the presence or absence of TGF-β1 (5 ng/ml) for 48 hours and followed by co-incubation with Dp44mT (10 μM) for another 24 h. The expression of indicated proteins were detected by immunoblotting. (C) Iron (FeCl_3_) inhibits Dp44mT's ability to attenuate the TGF-β1-induced EMT. HCC cells were pre-treated in the presence or absence of TGF-β (5 ng/mL) for 48 h and followed by co-incubation with either: FeCl_3_ (20 μM), Dp44mT (10 μM) or Dp44mT (10 μM) + FeCl_3_ (20 μM) for another 24 h. (D) Immunoblotting showed that TGF-β1 or shNDRG2 treatment increases the expression of Snail, Slug and pSMAD3. Dp44mT or NDRG2 overexpression can attenuate the effect of TGF-β1. β-actin was used as the internal control. All assays were done in triplicate.

TGF-β1 is known to play pivotal roles in promoting tumor cell invasion and metastasis and is overexpressed in advanced cancers including HCC.[26-28] We therefore examined whether Dp44mT could act against the TGF-β1- induced EMT in MHCC-97L and SMMC-7721 cells. TGF-β1 and shNDRG2 significantly enhanced their invasive capacity, and Dp44mT and NDRG2 overexpression attenuated the TGF-β1-induced promotion of invasion in MHCC-97L and SMMC-7721 cells (Fig. 4A). As shown by immunoblotting studies in Figure 4B, TGF-β1 markedly reduced E-cadherin levels in both MHCC-97L and SMMC-7721 cells. And NDRG2 knock-down mimics the TGF-β1-induced EMT in both MHCC-97L and SMMC-7721 cells. However, after Dp44mT treatment, the TGF-β1-induced reduction of E-cadherin was clearly attenuated. In agreement with the ability of Dp44mT to attenuate the TGF-β1-induced reduction of E-cadherin, Dp44mT also significantly decreased the TGF-β1-induced up-regulation of the N-cadherin and vimentin, in MHCC-97L and SMMC-7721 cells. Similarly, NDRG2 overexpression can attenuate the TGF-β1-induced EMT in MHCC-97L and SMMC-7721 cells. Supporting Information Figure 1C showed the expression of Flag and NDRG2 after transfection.

To demonstrate the importance of iron-depletion on the ability of Dp44mT to inhibit the TGF-β1-induced EMT, we further confirmed the phenomenon by co-incubating Dp44mT with iron (as FeCl_3_) to form iron complexes that cannot bind cellular iron. These complexes prevented the iron depletion-mediated up-regulation of NDRG2, but also inhibited the ability of Dp44mT to attenuate the TGF-β1-induced EMT, as shown by the analysis of E-cadherin, N-cadherin and vimentin expression (Fig. 4C). Together, these data demonstrated that Dp44mT can block the TGF-β1-induced EMT in MHCC-97L and SMMC-7721 cells and that this is dependent on chelation of cellular iron.

Next, we investigated the expression of Snail/Slug/pSMAD3, which were responsible for the regulation of E-cadherin. For Snail, Slug and pSMAD3, they were significantly increased after TGF-β1 or shNDRG2 treatment (Fig. 4D). Dp44mT or NDRG2 overexpression can attenuate the effect of TGF-β1 (Fig. 4D). Hence, Dp44mT or NDRG2 overexpression can repress the TGF-β/SMAD pathway.

### The gp130/STAT3 pathway plays a critical role in mediating Dp44mT Function

**Figure 5:**
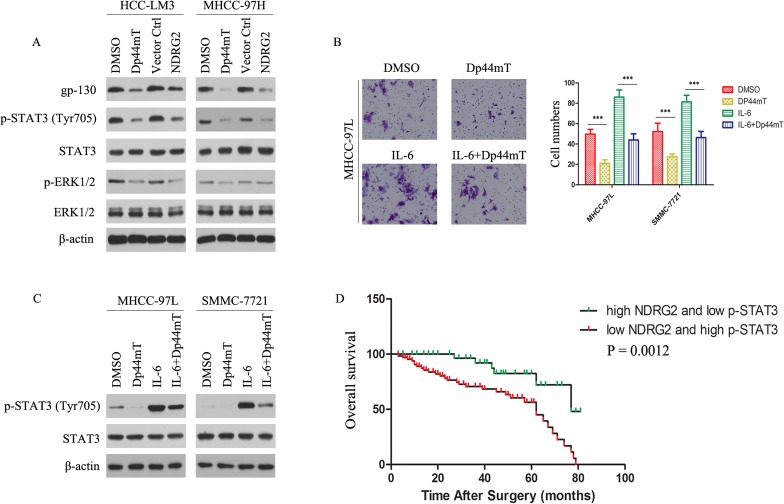
gp130/STAT3 pathway is involved in mediating Dp44mT action, and combination of NDRG2 expression and p-STAT3 signal is a powerful predictor of poor clinical outcome in HCCs (A) 24 hours after indicated treatment (Dp44mT 10 μM), HCC cells were analyzed for the indicated protein by immunoblotting. β-actin was used as the internal control. (B) Cell invasion assay showed Dp44mT attenuated the IL-6 (20 ng/ml) induced invasive capacity in MHCC-97L cells. The results represent means ± SD of experiments performed in triplicate. (C) Dp44mT reduced IL-6-induced phosphorylation of STAT3 in MHCC-97L and SMMC-7721 cells. β-actin was used as the internal control. (D) Combination of NDRG2 and p-STAT3 enhanced correlation to clinical parameters and the significance for poor prognosis.

Signaling pathway mediated by Dp44mT was analyzed by expression of gp130 and phosphorylated forms of STAT3. We first evaluated the effect of Dp44mT on the expression of gp130 and p-STAT3 in HCC-LM3 and MHCC-97H cells. Figure 5A showed that treatment of HCC-LM3 and MHCC-97H cells with Dp44mT for 24 h led to a significant reduction in gp130 expression as well as tyrosine-phosphorylated STAT3 although total STAT3 was unaffected. On the other hand, Dp44mT also caused decrease in p-ERK1/2 expression. While NDRG2 overexpressed in HCC-LM3 and MHCC-97H cells, the expression of gp130, p-STAT3 and p-ERK1/2 was also observed to be significantly decreased (Fig. 5A). Next, we examined whether Dp44mT could inhibit IL-6-induced STAT3 phosphorylation in MHCC-97L and SMMC-7721 cell lines. MHCC-97L and SMMC-7721 cells were pretreated with Dp44mT for 24 h and then stimulated with IL-6 (20 ng/ml) for 15 min. As shown in Figure 5C, IL-6 induced STAT3 phosphorylation was reduced by Dp44mT. Importantly, Dp44mT also can inhibit exogenous IL-6-induced invasion in MHCC-97L and SMMC-7721 cell lines (Fig. 5B). The results indicated that gp130/STAT3 pathway is likely an important target of Dp44mT in HCC cells.

### Combination of NDRG2 and p-STAT3 levels has better prognostic value for HCC

We further analyzed the expression levels of NDRG2 and p-STAT3 in clinical HCC samples through immunohistochemistry analysis. We analyzed 136 patient specimens. The results revealed patients whose tumors expressed below-average level of NDRG2 or above-average level of p-STAT3 exhibited significantly decreased trend in OS due to HCC-related death (Supporting Information Fig. 5A-B). For patients whose tumors had both below-average level of NDRG2 and above-average level of p-STAT3, adverse outcomes were exacerbated (Fig. 5D). Using the combination of these two parameters increased the prognostic value, as compared to using NDRG2 or p-STAT3 alone (Fig. 5D; Supporting Information Fig. 5A-B). In conclusion, evaluation of both NDRG2 expression and p-STAT3 signal is a powerful predictor of poor prognosis, further supporting loss of NDRG2 activated gp130/STAT3 signaling, resulting in EMT occurrence and thus metastases of HCC cells.

## DISCUSSION

HCC is the most common liver malignancy and a major health problem globally. Current standard practices for treatment of HCC, surgical resection, and liver transplantation are less than satisfactory due to metastasis and high recurrence rates.[29] Previous studies showed that iron homeostasis and iron regulatory pathways are abnormal in human HCC.[30-32] Thus, it is likely that iron chelators targeting excess iron may suppress HCC and bring clinical benefits to HCC patients. Some studies demonstrated that iron chelators are effective in HCC treatment.[33, 34] However, studies of the anti-metastasis of iron chelators for HCC have not been reported in either preclinical or clinical studies. In this study, we focused on the anti-metastatic potency against HCC of iron chelators.

It has been well documented that a number of cancers can be inhibited by iron chelators, particularly solid tumors, both *in vitro* and *in vivo*, where the agent has shown marked and selective efficacy with little toxicity to normal cells and tissues.[14, 15, 35] In this study, we demonstrated Dp44mT, a novel iron chelator, showed efficient anti-metastasis effect on human HCC, both *in vitro* and *in vivo*. The effect of Dp44mT on inhibiting the metastasis of HCC was attributed to its ability to markedly up-regulate NDRG2, a well-known anti-metastatic gene.[23, 36] The NDRG2 content was high in normal hepatocytes, decreased in noninvasive and primary HCC cells, and reached the lowest level in invasive HCC cells. This progressively decreased expression profile paralleled with deterioration of the disease, suggesting a role of NDRG2 in progression of HCC.

The effect of Dp44mT on HCC invasion and metastasis was directly demonstrated in our *in vitro* and *in vivo* studies. In orthotopic xenografts, Dp44mT group generated fewer lung metastasis foci, indicating Dp44mT inhibited aggressive and metastatic properties of HCC. Moreover, up-regulation of NDRG2 led to severe suppression of lung metastasis of HCC in mice. To our knowledge, this is the first report that NDRG2 is critical for Dp44mT to inhibit HCC metastasis, in addition to suppress tumor proliferation and growth.

In HCC, tumor cells grow embedded in a microenvironment with a high content of extracellular matrix (ECM) proteins, such as laminin, collagen, vitronectin and fibronectin, as a consequence of the development of cirrhosis.[37] Therefore, crossing the ECM barriers by tumor cells is regarded to be particularly important for metastatic progression of HCC. Among the many different categories of molecules regulating tumor metastasis, we focused on the effect of Dp44mT on the expression of the genes responsible for tumor invasion such as EMT regulators and ECM-degrading enzymes. Membrane E-cadherin, N-cadherin and vimentin are important hallmarks of the EMT. Gelatinases (MMP2) are important players of tumor invasion that degrade ECM components and activate the latent TGF-β1 present in extracellular space.[18, 38] We demonstrated that incubation of HCC-LM3 and MHCC-97H cells with Dp44mT led to the accumulation of E-cadherin and loss of N-cadherin and vimentin. We also found that Dp44mT can antagonize the tumor invasion and by abrogating the induction of gelatinase activity (MMP2).

We further demonstrated that incubation of MHCC-97L and SMMC7721 cells with TGF-β1 led to the reduction of E-cadherin and accumulation of N-cadherin and vimentin, which was consistent with the EMT. We further showed that Dp44mT could inhibit the TGF-β1-induced reduction of E-cadherin at the membrane and that this could be mediated by NDRG2. Indeed, NDRG2 overexpression could maintain membrane localization of E-cadherin, and incubation with iron chelators led to the same result. Conversely, NDRG2 knock-down caused a loss of membrane E-cadherin with some nuclear translocation in MHCC-97L and SMMC-7721 cells. And NDRG2 overexpression or Dp44mT also inhibited the TGF-β/SMAD pathway, which can activate the EMT in cancer cells. So our results suggested that NDRG2 may play important roles in Dp44mT antagonized the invasion-promoting activity of TGF-β1 during metastasis. We propose that these results might apply to a number of additional cancer types other than HCC because NDRG2 is frequently downregulated in many other cancer types as well.[19-23]

A increase in gp130 signal and activation of STAT3 is a key tumor survival mechanism, and promotes tumor metastatic processes including EMT and resistance to apoptosis.[39, 40] Previous studies have demonstrated that activated STAT3 plays a critical role in hematogenous intrahepatic metastasis in an orthotopic implantation model of HCC.[41]

Our current *in vitro* and *in vivo* studies suggested that NDRG2/gp130/STAT3 signal way was responsible for Dp44mT-mediated inhibition of invasion/metastasis. Furthermore, we found that NDRG2 decreased the expression of gp130 and negatively regulated the activation of STAT3. Given complex p-STAT3 pathways, whether other upstream regulators are involved in Dp44mT-inhibiting p-STAT3 signal remains to be further determined. Remarkably, the predictive range of NDRG2 expression levels combined with p-STAT3 signal was more sensitive than that of NDRG2 alone for OS, strongly suggesting that the concerted activities of NDRG2 and p-STAT3 detected in our experiments are recapitulated in clinical patients with HCC. Combined evaluation of NDRG2/p-STAT3 level as a new prognostic marker in patients with HCC is important because they provide not only a new criterion for prognosis, but also a potential therapeutic target.

In summary, we have demonstrated that Dp44mT mediates multiple facets essential for HCC development and metastasis. In particular, the data has led us to propose that combination of NDRG2 with p-STAT3 is a novel marker in the prognosis of HCC and a potential therapeutic target. Because NDRG2 is also decreased in other types of cancers,[19-23] we believe that Dp44mT can be widely used in human cancers to inhibit proliferation and prevent metastasis.

## MATERIALS AND METHODS

### Patients and samples

136 patients were randomly retrieved from HCC patients who underwent curative resection in The First Affiliated Hospital of Harbin Medical University (Harbin, China) from March 2007 to July 2010. The detailed clinicopathologic characteristics of the patients are listed in Table 1.These HCC patients were monitored after surgery, until September, 2013. All specimens were collected immediately after resection, and fixed in 10% formalin. None of the patients received any preoperative anticancer treatment. Curative resection was defined as: (i) the complete resection of all tumor nodules and the cut surface being free from cancer on histological examination; (ii) no cancerous thrombus found in the portal vein (main trunk or two major branches), hepatic veins or bile duct; (iii) no extrahepatic metastasis and (iv) negative serology and imaging studies at 2 months after operation. Overall survival (OS) was defined as the interval between the dates of surgery and death.

In addition, 15 normal liver (which were obtained from patients with hemangiomas of liver who underwent surgery), 15 non-invasive HCC (without vascular invasion) and 15 invasive HCC (with vascular invasion) fresh samples were also obtained from The First Affiliated Hospital of Harbin Medical University. Patient samples were obtained following informed consent according to an established protocol approved by the Ethic Committee of The First Affiliated Hospital of Harbin Medical University.

### Cell lines and cell treatments

Normal liver cell lines HL-7702, QSG-7701 and liver cancer cell lines SMMC-7721, MHCC-97L, MHCC-97H and HCC-LM3 were purchased from Cell Bank of Type Culture Collection of Chinese Academy of Sciences, Shanghai Institute of Cell Biology, Chinese Academy of Sciences. Cell lines were maintained at 37 °C in a humidified incubator containing 5% CO_2_, in Dulbecco's Modified Eagle Medium (Gibco, Invitrogen Company, Grand Island, NY) supplemented with 10% fetal bovine serum (Gibco) and 1% penicillin/streptomycin (Gibco). provider. Human recombinant TGF-β1 was obtained from R&D Systems (Minneapolis, MN) and used at a final concentration of 5 ng/ml. The cells were incubated in serum-free medium overnight, and then treated with TGF-β1 for 48 h. Dp44mT and IL-6 were purchased from Sigma (St. Louis, MO). The chelator Dp44mT was utilized at a low concentration since this ligand shows far higher membrane permeability and demonstrates marked iron chelation efficacy.[15] Dp44mT was freshly dissolved in DMSO (Sigma) and diluted in culture media (final [DMSO]: ≤ 0.1% (v/v)).

### Retroviral infection and lentiviral infection

Human pLPCX-NDRG2-Flag and pGIPZ-shNDRG2 were purchased from Biowot Technologies (Shenzhen, China). To generate retrovirus, the packaging line GP2-293T was cotransfected with pCMV-VSVG, and one of either pLPCX-Control or pLPCX-NDRG2-Flag, using FuGENE 6 Transfection Reagent (Roche Diagnostics Corp., Indianapolis, IN). To generate lentivirus, the packaging line 293T was cotransfected with psPAX2, pMD2.G and one of either pGIPZ-shControl or pGIPZ-shNDRG2, using FuGENE 6 Transfection Reagent (Roche Diagnostics Corp., Indianapolis, IN). Retrovirus- or lentivirus-containing conditioned medium was harvested, filtered through a 0.45 μm membrane, and used to transduce HCC cells according to standard procedures. Following retroviral or lentiviral infection, single-cell clonal isolates were selected in the presence of puromycin for 2 to 4 weeks.

### Immunoblot analysis

For preparing total cell lysates, cells were lysed in lysis buffer (Invitrogen), incubated on ice for 30 min and centrifuged for 20 min to remove cell debris. Total cell lysate was subjected to SDS–polyacrylamide gel electrophoresis. The proteins were then electro-transferred to polyvinylidene difluoride membrane (Millipore, Billerica, MA) and incubated overnight with antibodies at 4°C. Subsequently, the membranes were incubated with secondary antibodies for 1 hour at room temperature and the signal was detected using an enhanced chemiluminescence detection kit (Pierce, Rockford, IL). The primary antibodies: NDRG2, E-cadherin, N-cadherin and vimentin were purchased from Abcam (Cambridge, MA). TWIST1 and gp-130 were purchased from Santa Cruz Biotechnology (Dallas, Texas). STAT3, p-STAT3 (Tyr705), ERK1/2 and p-ERK1/2 were purchased from Cell Signaling Technology (Danvers, MA). Flag and β-actin was purchased from Sigma. The secondary antibodies, anti-mouse IgG-HRP and anti-rabbit IgG-HRP were purchased from Santa Cruz Biotechnology.

### Detection of MMP-2 activity by Gelatin Zymography

After treatment, the medium was collected to detect the protein concentrations and were loaded onto zymographic sodium dodecyl sulfate gel containing gelatin (1 mg/mL). Then the gel was incubated in renaturing buffer and developing buffer (invitrogen) according to the manufacturer's instructions. The enzyme activity was visualized by staining the gel with Coomassie Blue R-250 (Sigma).

### Immunofluorescence

Briefly, cells seeded on coverslips were fixed with 4% (w/v) paraformaldehyde (Sigma) for 10 min and permeabilized with 0.1% (v/v) Triton X-100 for 5 min at room temperature. The cells were then incubated overnight with primary antibodies at 4 °C, followed by incubation with fluorescent secondary antibody (invitrogen) for 1 hour at room temperature. After final washes with PBS, the coverslips were mounted using an anti-fade mounting solution containing 4',6-diamidino-2-phenylindole (DAPI; Vector lab, Burlingame, CA) and images were examined and captured.

### Cell invasion and adhension assays

Invasion was measured by using 24-well BioCoat cell culture inserts (BD Biosciences, NJ) with an 8 μm–porosity polyethylene terephthalate membrane coated with Matrigel Basement Membrane Matrix. For adhesion assay, we used the CytoSelect^TM^ Cell Adhesion Assay Kit (Cell Biolabs, San Diego, CA). The adherent cells are extracted and measured the OD 560nm in a plate reader.

### Quantitative Taqman real-time PCR

Total RNA from cells or human tissues were prepared with RNeasy kit (Qiagen, Valencia, CA). cDNA was prepared by using TaqMan® Reverse Transcription Reagents (Applied Biosystems, Grand Island, NY). Human NDRG2 probe and β-actin probe were purchased from Applied Biosystems. Taqman real-time PCR was done with TaqMan PCR mixture (Applied Biosystems). The other genes were analyzed using SYBR Green PCR mixture (Applied Biosystems). The primer sequences are provided as following:

**Table:** 

Gene	Forward Primer	Reverse Primer
E-cadherin	5′-TGCCCAGAAAATGAAAAAGG-3′	5′-GTGTATGTGGCAATGCGTTC-3′
N-cadherin	5′-AGGATCAACCCCATACACCA-3′	5′-TGGTTTGACCACGGTGACTA-3′
Vimentin	5′-GAGAACTTTGCCGTTGAAGC-3′	5′-TCCAGCAGCTTCCTGTAGGT-3′
TWIST1	5′-GGAGTCCGCAGTCTTACGAG-3′	5′-TCTGGAGGACCTGGTAGAGG-3′
Cytokeratin-8	5′-CAGGAGCTGATGAACGTCAA-3′	5′-TCCAGCAGCTTCCTGTAGGT-3′
Cytokeratin-17	5′-CGGAGACAGAGAACCGCTAC-3′	5′-CACAATGGTACGCACCTGAC-3′
Cytokeratin-18	5′-CACAGTCTGCTGAGGTTGGA-3′	5′-GAGCTGCTCCATCTGTAGGG-3′
Claudin-1	5′-AATTTTCATCGTGGCAGGTC-3′	5′-AGGACAGGAACAGGAGAGCA-3′
Claudin-8	5′-GGCTGTTTCTTGGTGGTGTT-3′	5′-TCTCCACTGAGGCATGACAG-3′
Jagged-1	5′-GTCCCACTGGTTTCTCTGGA-3′	5′-CCACAGACGTTGGAGGAAAT-3′
Jagged-2	5′-GTCAAGGTGGAGACGGTTGT-3′	5′-TTGCACTGGTAGAGCACGTC-3′
Goosecoid	5′-GCTTTTCGTGCAGAACCAGT-3′	5′-CGGTTCTTGAACCAGACCTC-3′
β-actin	5′-CGCGAGAAGATGCCCAGATC-3′	5′-TCACCGGAGTCCATCACGA-3′

Real-time PCR was done in triplicate. The expression of genes was normalized to the β-actin gene.

### Immunohistochemistry analysis

Immunohistochemistry was performed as described previously[42] using NDRG2, E-cadherin, N-cadherin and vimentin antibodies. In brief, tissue sections were deparaffinized in xylene and rehydrated with ethanol. Tissue sections were then preincubated with 10% normal goat serum in PBS (pH 7.5) followed with incubation with primary antibody overnight at 4°C. Tissue sections were then stained with biotinylated secondary antibody (Vector lab) for 1 hour at room temperature, followed by the Vectastain Elite ABC reagent (Vector lab) for 30 min. The peroxidase reaction was developed with diaminobenzidine (DAB kit; Vector lab) and the slides were counterstained with hematoxylin (Sigma).

### Nude mice xenograft and orthotopic model studies

All animal experiments met the requirement of Harbin Medical University Animal Care Facility and the National Institutes of Health guidelines. For assessment the effect of Dp44mT in orthotopic tumor xenografts, an orthotopic liver tumor model in nude mice with higher potential of distant (lung) metastases was established. Briefly, we infected HCC-LM3 cells with lentiviral-shNDRG2 or lentiviral-shCtrl. And we infected MHCC-97H cells with retrovial-NDRG2 or retrovial-Ctrl. Then these approximately 1×10^7^ infected or mock cells in 0.2 ml culture medium phosphate buffered saline (PBS) were injected subcutaneously into the right flank of the mice, which were then observed daily for signs of tumor development. Once the subcutaneous tumor reached 1-1.5 cm in diameter, it was removed and cut into about 1-2 mm^3^ cubes which were implanted into the left liver lobe of another group of nude mice. A week later, the nude mice were treated with Dp44mT (0.4 mg/kg). Dp44mT was dissolved in 30% propylene glycol in 0.9% saline and injected intravenously (via the tail vein) 5 days/week (Monday to Friday). Liver tumors and lung tissues were harvested 4 weeks after Dp44mT treatment. The lung metastases were confirmed by H&E staining. All experiments were performed with at least 6 mice in each group, and all of the experiments were repeated 3 times.

### Statistic analysis

Statistical analysis was performed with the GraphPad Prism software package (v. 4.02; San Diego, CA) or SPSS 16.0 software (Chicago, IL), and *P* < 0.05 was considered statistically significant.

## ABBREVIATIONS

NDRG2: downstream-regulated gene 2
HCC: hepatocellular carcinoma
EMT: epithelial-mesenchymal transition
DFO: desferrioxamine
Dp44mT: di-2-pyridylketone-4,4-dimethyl-3-thiosemicarbazone
DpT: di-2-pyridyl thiosemicarbazone
CRC: colorectal cancer
OS: overall survival
